# Adjuvant corticosteroid therapy for critically ill patients with COVID-19

**DOI:** 10.1186/s13054-020-02964-w

**Published:** 2020-05-19

**Authors:** Xiaofan Lu, Taige Chen, Yang Wang, Jun Wang, Fangrong Yan

**Affiliations:** 1 State Key Laboratory of Natural Medicines, Research Center of Biostatistics and Computational Pharmacy, China Pharmaceutical University, Nanjing, 210009 China; 2 Medical School of Nanjing University, Nanjing, China; 3 Department of Radiology, The Affiliated Nanjing Drum Tower Hospital of Nanjing University Medical School, Nanjing, China; 4 Department of Intensive Care Medicine, The First Affiliated Hospital of Soochow University, Suzhou, China

Dear Editor,

A substantial portion of patients with coronavirus disease (COVID-19) developed rapidly progressive pneumonia leading to acute respiratory distress syndrome (ARDS) and multiple organ dysfunctions, conditions associated with high mortality [1]. Adjuvant corticosteroid therapy of such patients is common in clinical practice, but evidence is scarce regarding the efficacy of adjuvant corticosteroids in patients who are critically ill with COVID-19.

We retrospectively reviewed medical records of adult patients with COVID-19 who were admitted to Tongji Hospital (Wuhan, China) from January 25 to February 25, 2020. Two hundred forty-four eligible patients who had complete records and were critically ill and treated with antiviral agents were enrolled. Critically ill patients were defined as those with ARDS (PaO_2_/FiO_2_ ≤ 300 mmHg; when PaO_2_ is not available, SpO_2_/FiO_2_ ≤ 315 suggests ARDS) or sepsis with acute organ dysfunction [2]. We converted all preparations to hydrocortisone-equivalent doses (methylprednisolone 1:5, dexamethasone 1:25) [3]. The clinical outcome was 28-day mortality after admission.

We adjusted for differences in baseline characteristics by propensity score, using multivariate logistic regression without regard to outcomes [4]. Potential confounders considered in propensity score matching (PSM) were variables included in the final model by step-wise backward elimination with *P* < 0.20 [5]. Corticosteroid treatment effect on outcome was analyzed by multivariate logistic regression with adjustment for major variables (age, SpO_2_/FiO_2_, and lymphocytes) associated with mortality; individual propensity score was incorporated as a covariable to calculate the propensity-adjusted odds ratio (OR) [5]. PSM generated propensity score-matched pairs without replacement, and survival probability was compared by the Kaplan-Meier curve and analyzed with the log-rank test. Cox regression was used to estimate hazard ratio (HR) with 95% CI. For unadjusted comparisons, a two-sided *P* < 0.05 was considered statistically significant.

Of the 244 critically ill patients with COVID-19, the median age was 62 (50–71) years, and 52% were male. All patients were given antiviral therapy (e.g., oseltamivir, arbidol, lopinavir/ritonavir, ganciclovir, interferon-α), and 151 (62%) were given adjuvant corticosteroid treatment (median hydrocortisone-equivalent dosage 200 [range 100–800] mg/day). Five (5.4%) and 79 (52.3%) patients died in non-steroid and steroid groups, respectively. The median (IQR) administration duration of corticosteroid treatment was 8 (4–12) days. Multiple organ dysfunctions were more common in the steroid group than in the non-steroid group. Multivariate analysis that adjusted for major mortality-associated variables and propensity score indicated that corticosteroid treatment was independent from overall mortality (adjusted OR 1.05; 95% CI 0.15–7.46). One hundred forty-seven (60%) had dyspnea and 87 (36%) had ARDS, and subgroup analyses revealed corticosteroid treatment was not associated with 28-day mortality (both, *P* > 0.3). Sixty-two patients in 31 pairs were matched (Table 1), and 28-day mortality rate was 39% in case subjects and 16% in control subjects (*P* = 0.09). Likewise, addition of adjuvant corticosteroid therapy to standard antiviral treatment was not associated with 28-day mortality (*P* = 0.17; Fig. 1). However, increased corticosteroid dosage was significantly associated with elevated mortality risk after adjustment for administration duration (*P* = 0.003); every 10-mg increase in dosage was associated with additional 4% mortality risk (adjusted HR 1.04, 95% CI 1.01–1.07).

**Table 1:** Baseline characteristics for steroid treatment and non-steroid treatment groups comprising critically ill patients with COVID-19 before and after propensity score matching

	Cohort study			Case-control study (PSM)		
	Steroid (151)	Non-steroid (93)	*P*	Steroid (31)	Non-steroid (31)	*P*
Age, years	64 (53–71)	59 (47–69)	.09	57 (51–69)	58 (50–67)	.98
Gender, male	83 (55)	45 (48)	.39	16 (52)	16 (52)	1
Signs and symptoms						
Fever	136 (90)	81 (87)	.61	30 (97)	26 (84)	.2
Dry cough	112 (74)	57 (61)	.05	21 (68)	21 (68)	1
Dyspnea	94 (62)	53 (57)	.5	20 (65)	19 (61)	1
Fatigue	70 (46)	48 (52)	.51	12 (39)	13 (42)	1
Expectoration	69 (46)	30 (32)	.05	12 (39)	12 (39)	1
Diarrhea	45 (30)	22 (24)	.37	10 (32)	7 (23)	.57
Anorexia	42 (28)	25 (27)	.99	5 (16)	8 (26)	.53
Original comorbidities						
Hypertension	61 (40)	34 (37)	.64	16 (52)	12 (39)	.44
Diabetes	34 (23)	10 (11)	.03	4 (13)	7 (23)	.51
CVD	15 (10)	13 (14)	.45	2 (7)	2 (7)	1
COPD	9 (6)	3 (3)	.51	0	1 (3)	1
Vital signs						
T, °C	37.0 (36.2–38)	36.7 (36.4–37.3)	.02	37 (36.5–37.6)	37 (36.5–37.3)	.93
Breathing, rpm	22 (20–25)	20 (20–22)	< .01	21 (20–24)	20 (20–22)	.08
Pulse, bpm	92 (82–105)	88 (78–98)	.03	95 (78–106)	93 (82–100)	.51
SpO_2_/FiO_2_	259 (121–303)	297 (279–388)	< .01	291 (212–452)	294 (246–396)	.57
Laboratory findings (WBCs, lymphocytes, neutrophils, platelets, × 10^9^/L)						
WBCs	6.7 (4.9–8.9)	5.0 (4.0–6.5)	< .01	6.6 (4.0–8.6)	5.1 (3.5–6.8)	.12
Lymphocytes	0.7 (0.5–1.0)	1.2 (0.9–1.6)	< .01	0.9 (0.5–1.3)	1.1 (0.6–1.2)	.64
Neutrophils	5.4 (3.6–7.6)	3.2 (2.4–4.2)	< .01	5.2 (2.6–7.4)	3.5 (2.3–4.7)	.09
Platelets	181 (138–248)	224 (170–298)	< .01	168 (138–214)	206 (155–230)	.23
HGB, g/L	130 (117–141)	127 (117–139)	.42	128 (118–138)	125 (117–133)	.56
Organ function damage						
ARDS	81 (54)	6 (7)	< .01	12 (39)	6 (19)	.16
Septic shock	69 (46)	2 (2)	< .01	8 (26)	2 (7)	.08
Myocardial infarction	64 (42)	3 (3)	< .01	10 (32)	3 (10)	.06
AKI	46 (31)	5 (5)	< .01	8 (26)	3 (10)	.18
DIC	39 (26)	2 (2)	< .01	6 (19)	2 (7)	.26
Liver injury	28 (19)	6 (7)	< .01	7 (23)	3 (10)	.3
Treatment						
Anti-bacteria	142 (94)	42 (45)	< .01	25 (81)	26 (84)	1
Gamma globulin	84 (56)	8 (9)	< .01	11 (36)	8 (26)	.58
MV	78 (52)	4 (4)	< .01	11 (36)	4 (13)	.08
Muscle relaxant	25 (17)	0	< .01	4 (13)	0	.12
HFNC	21 (14)	1 (1)	< .01	6 (19)	1 (3)	.11

*Abbreviations*: *CVD* cardiovascular disease, *COPD* chronic obstructive pulmonary disease, *WBCs* white blood cells, *ARDS* acute respiratory distress syndrome, *AKI* acute kidney injury, *DIC* disseminated intravascular coagulation, *MV* mechanical ventilation, *HFNC* high flow nasal cannula

Continuous variables were described as the median (IQR) while categorical variables were expressed as frequencies (%). Hypothesis testing using Fisher’s exact test for categorical data and Mann-Whitney test for continuous data

**Fig. 1:**
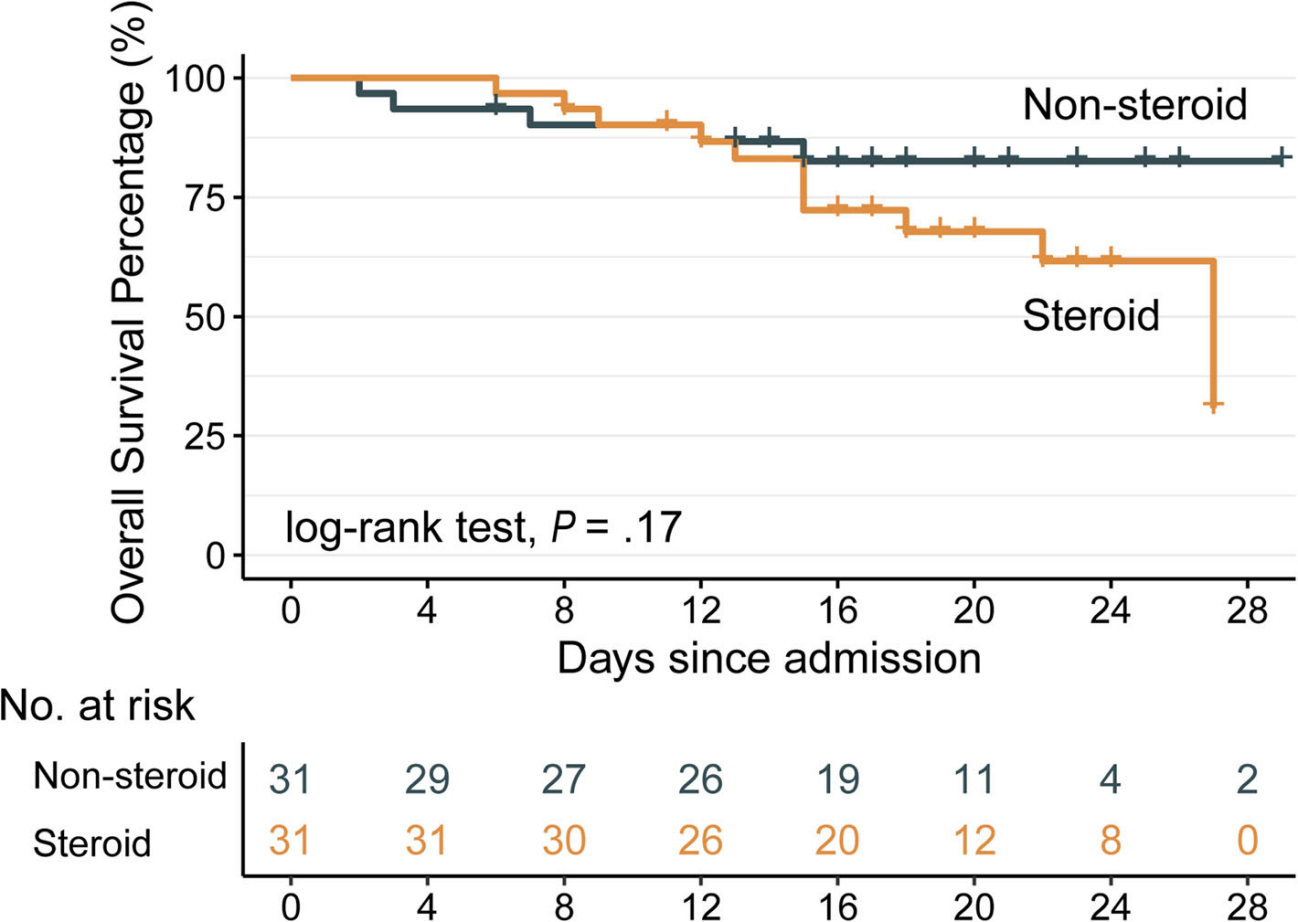
Survival curves stratified by adjuvant corticosteroid treatment. Thirty-one critically ill patients with COVID-19 who received corticosteroid treatment (yellow line) are compared with 31 matched control subjects (green line) who did not receive corticosteroid treatment

We acknowledged limitations. First, we did not distinguish patients who received corticosteroids for underlying disease (e.g., COPD), the number of which was however small. Second, PSM is limited by adjusting for observed covariables only; randomized placebo-controlled trials are therefore warranted. Altogether, our investigation indicated limited effect of corticosteroid therapy could pose to overall survival of critically ill patients with COVID-19. Given the adverse effects, corticosteroid therapy must be commenced with caution, and prudent dosage should be promoted under certain circumstances.

## Data Availability

Dr. J. Wang had full access to all of the data in the study. After publication, the data will be made available to others on reasonable requests after approval from the corresponding author (J.W, dr_wangjun@suda.edu.cn) and Wuhan Tongji Hospital.
